# Tumor treating fields (TTFields) delay DNA damage repair following radiation treatment of glioma cells

**DOI:** 10.1186/s13014-017-0941-6

**Published:** 2017-12-29

**Authors:** Moshe Giladi, Mijal Munster, Rosa S. Schneiderman, Tali Voloshin, Yaara Porat, Roni Blat, Katarzyna Zielinska-Chomej, Petra Hååg, Ze’ev Bomzon, Eilon D. Kirson, Uri Weinberg, Kristina Viktorsson, Rolf Lewensohn, Yoram Palti

**Affiliations:** 1 Novocure Ltd, 31905 Haifa, Israel; 2 Department of Oncology-Pathology, Karolinska Institutet, SE-171 76 Stockholm, Sweden

**Keywords:** TTFields, Radiation treatment, Glioma, Radiosensitization, DNA damage repair

## Abstract

**Background:**

Tumor Treating Fields (TTFields) are an anti-neoplastic treatment modality delivered via application of alternating electric fields using insulated transducer arrays placed directly on the skin in the region surrounding the tumor. A Phase 3 clinical trial has demonstrated the effectiveness of continuous TTFields application in patients with glioblastoma during maintenance treatment with Temozolomide. The goal of this study was to evaluate the efficacy of combining TTFields with radiation treatment (RT) in glioma cells. We also examined the effect of TTFields transducer arrays on RT distribution in a phantom model and the impact on rat skin toxicity.

**Methods:**

The efficacy of TTFields application after induction of DNA damage by RT or bleomycin was tested in U-118 MG and LN-18 glioma cells. The alkaline comet assay was used to measure repair of DNA lesions. Repair of DNA double strand breaks (DSBs) were assessed by analyzing γH2AX or Rad51 foci. DNA damage and repair signaled by the activation pattern of phospho-ATM (pS1981) and phospho-DNA-PKcs (pS2056) was evaluated by immunoblotting. The absorption of the RT energy by transducer arrays was measured by applying RT through arrays placed on a solid-state phantom. Skin toxicities were tested in rats irradiated daily through the arrays with 2Gy (total dose of 20Gy).

**Results:**

TTFields synergistically enhanced the efficacy of RT in glioma cells. Application of TTFields to irradiated cells impaired repair of irradiation- or chemically-induced DNA damage, possibly by blocking homologous recombination repair. Transducer arrays presence caused a minor reduction in RT intensity at 20 mm and 60 mm below the arrays, but led to a significant increase in RT dosage at the phantom surface jeopardizing the “skin sparing effect”. Nevertheless, transducer arrays placed on the rat skin during RT did not lead to additional skin reactions.

**Conclusions:**

Administration of TTFields after RT increases glioma cells treatment efficacy possibly by inhibition of DNA damage repair. These preclinical results support the application of TTFields therapy immediately after RT as a viable regimen to enhance RT outcome. Phantom measurements and animal models imply that it may be possible to leave the transducer arrays in place during RT without increasing skin toxicities.

**Electronic supplementary material:**

The online version of this article (10.1186/s13014-017-0941-6) contains supplementary material, which is available to authorized users.

## Background

Along with surgical resection or diagnostic biopsy, chemotherapy with temozolomide (TMZ) and radiation treatment plays a key role in the treatment of glioblastoma (GBM). Advances with radiation treatment (RT) have been achieved through successfully combining this modality with conventional chemotherapeutic agents [1–6]. However, combining RT and chemotherapy rarely leads to long-term survival for GBM patients. While there are incremental efficacy benefits when TMZ is added to RT, there are also cumulative systemic toxicities that limit the doses that can safely be delivered [7]. Thus, there is a need to develop therapeutic strategies that can enhance RT efficacy without incurring additional systemic toxicity.

Tumor-treating fields (TTFields) are a proven therapeutic modality delivered via non-invasive application of low-intensity (1-3 V/cm), intermediate-frequency (100-500 kHz), alternating electric fields. A phase 3 clinical trial has demonstrated the effectiveness and safety of continuous TTFields application in patients with GBM when applied during maintenance treatment with TMZ [7]. TTFields therapy is given via opposing insulated ceramic transducer arrays that are attached to the skin in the region of the tumor. A layer of conductive hydrogel is placed between the ceramic transducers array and the skin, to maintain good conductance. TTFields therapy has a good safety profile with the main adverse event associated with TTFields treatment being skin irritation below the transducer arrays [7–11].

In cells undergoing mitosis, TTFields are thought to generate electric forces that cause dielectrophoresis and dipole alignment, leading to aberrant mitosis and subsequent cell death [12]. TTFields disrupt spindle structure through microtubule depolymerization and inhibit the mitotic Septin complex localization to the anaphase spindle midline, thereby preventing normal segregation of chromosomes and cytokinesis [12–16].

Kim et al. recently reported on a synergistic enhancement of RT cellular response when TTFields were given prior to RT [17]. They show that TTFields administered before RT caused blockade of DNA repair, increased mitotic catastrophe and decreased glioma cell survival. Hence, the results from Kim et al. support the hypothesis that TTFields may be used as an anti-mitotic modality and could also be combined with RT to sensitize glioma cells to treatment**.**

In the current study, we utilized a pair of glioma cell lines to investigate the outcome of TTFields application after the induction of DNA damage due to RT exposure. In order to distinguish between anti-mitotic effects and possible inhibition of DNA damage response (DDR) due to the effects of TTFields, we also examined the impact of delaying TTFields application after RT. Extrapolating the combination of TTFields with RT into the clinical setting would require either leaving the transducer arrays on the patients’ skin during the course of RT radiotherapy or removal of the arrays prior to irradiation. Therefore, we also investigated the absorptive properties of the ceramic transducers and how the transducer system influenced the irradiation effects on rat skin.

## Methods

### Cell culture

Glioma cell lines: U-118 MG (HTB-15) and LN-18 were obtained from the American Type Culture Collection (ATCC Manassas, VA, USA). Cells were resuscitated from early passage liquid nitrogen stocks and cultured less than 2 months before reinitiating cultures. Cells were maintained in Dulbecco’s modified Eagle’s medium supplemented with 10% (*v*/v) fetal bovine serum, 2 mM L-glutamine and penicillin/streptomycin (50 μg/ml). To inflict DNA damage, bleomycin (Calbiochem, San Diego, CA, USA) was added to cultures at one of the following concentrations: 0.4, 2, 10, 50 or 250 μg/ml.

### Cell irradiation

Cells were irradiated with doses of 0.5, 1, 2, 4, 6 or 8 Gy, using a 6 MV photon beam (Elekta Precise linear accelerator, Elekta Oncology Systems Stockholm, SWE) at a dose-rate of 0.25 Gy/min (Department of Radiation Therapy, Rambam Health Care Campus, Haifa, Israel) and kept on ice until TTFields application was initiated.

### TTFields application

TTFields were applied using the inovitro™ system (Novocure Haifa, ISR). In this system, two pairs of transducer arrays were printed perpendicularly on the outer walls of a Petri dish composed of a high dielectric constant ceramic (lead magnesium niobate–lead titanate [PMN-PT]). The transducer arrays were connected to a sinusoidal waveform generator that produced alternating electric fields (1.75 V/cm RMS, 200 kHz) in the medium. The orientation of the TTFields were switched 90° every 1 s. Temperature was measured by 2 thermistors attached to the ceramic walls. The efficacy of the combined treatment of TTFields and 4 Gy RT was tested following 72 h TTFields administration that was applied immediately after RT or 1 h, 4 h, or 24 h after RT.

### Cell viability

Inhibition of tumor cell growth was analyzed by quantification of cell numbers using Scepter 2.0 automated cell counter (EMD Millipore Billerica, Massachusetts, USA). The relative number of viable cells at the end of treatment was calculated as percentage of the untreated control.

### Clonogenic survival assay

Cells treated with RT, TTFields, and the RT/TTFields combination were subsequently harvested and re-seeded in 6-well tissue culture plates at a density of 300 cells/well. The resulting colonies (containing at least 50 cells) formed after 7 to 14 days were counted after staining with 0.5% crystal violet solution. The clonogenic survival was calculated relative to untreated cells. Cell viability and clonogenic effects were both measured at varying time points after treatments. Surviving fraction was calculated as product of cell viability at the end point of TTFields application and the clonogenic effect.

### Alkaline comet assay

Cellular DNA was stained with Syber green (Molecular Probes, Invitrogen) and the comets were analyzed with a 200× Nikon Eclipse TS100 microscope equipped with the Nikon Epi-fluoresence attachment (Nikon Melville, NY, USA). Images were captured with a Nikon DS-Fi2 camera (Nikon Melville, NY, USA). Image analysis was carried out with CometScore software (TriTek Wilmington, DE, USA). To quantify the remaining DNA breaks the percent of tail moment was calculated using the formula: (tail moment)/(tail moment at 1 h after RT or bleomycin) × 100.

### Immunofluorescence foci analyses

To assess DNA DSBs, cells grown on glass cover slips were irradiated with 4 Gy and treated with TTFields applied for 1 h, 2 h, 4 h, or 24 h. At these time points cells were fixed in freshly prepared 4% paraformaldehyde solution for 10 min. at room temperature. For staining, the fixed cells were permeabilized with 0.1% Triton X-100, and incubated with anti-γH2AX antibody (1:500) (Abcam Cambridge, GBR) or anti-Rad51 antibody (1:500) (Abcam Cambridge, GBR) at room temperature followed by incubation with secondary Cy-3 conjugated antibody (Jackson Immunoresearch West Grove, PA, USA). Slides were mounted in prolonged anti-fade solution supplemented with DAPI (Sigma Aldrich St. Louis, MO, USA). Images were collected using a LSM 700 laser scanning confocal system (Zeiss Göttingen, DEU), attached to an upright motorized microscope with a 63X/1.40 oil objective (ZeissAxio Imager Z2 Göttingen, DEU). Image analysis was carried out with Image J software (NIH, Maryland, USA).

### Extraction and western blot analyses of DNA-PKcs and ataxia telangiectasia mutated (ATM)

To assess DNA-PKcs phosphorylation and expression, cells were harvested in hypotonic buffer (1.5 mM MgCl2, 10 mM KCl, 0.5 mM DTT, 0.5% NP40, 10 mM HEPES pH 7.9) containing protease and phosphatase inhibitor cocktail (Sigma-Aldrich). The samples were centrifuged at 1000×g for 10 min at 4 °C to pellet nuclei, which were resuspended in RIPA buffer (50 mM Tris-HCl, 150 mM NaCl, 10 mM EDTA, 0.1% Na-Deoxycholate, 1% NP-40, 0.1% SDS) supplemented as above, sonicated and centrifuged at 20,000×g for 15 min. at 4 °C. The resulting supernatant, which contained solubilized proteins, were used for western blot analysis as indicated below.

For analyses of total and phosphorylated ATM (S1981), cells after treatment were harvested using pre-cooled PBS and RIPA buffer (50 mM Tris-HCl, 150 mM NaCl, 1 mM EDTA, 0.1% Na-Deoxycholate, 1% NP-40). Cells were scraped from the dishes and transferred into a pre-cooled microfuge tube. Cell suspension was agitated 30 min at 4 °C followed by centrifugation at 12,000×g for 20 min. at 4 °C from which the supernatant was collected for western blot analysis.

For western blot analysis, NuPAGE LDS Sample Buffer and NuPAGE Reducing Agent (Invitrogen Carlsbad, California, USA) were added to 30 μg of each protein sample and the mixtures were heated at 70 °C for 10 min. Proteins were resolved on 3-8% NuPAGE Novex Tris-Acetate gel (Invitrogen Carlsbad, California, USA) and transferred onto nitrocellulose membrane overnight at 30 V, 4 °C. The following antibodies were applied after blocking the membranes in 5% bovine serum albumin in TBST for 1 h: rabbit anti-pDNA-PKcs (S2056) (1 μg/ml; Abcam Cambridge, GBR) or rabbit anti-DNA-PKcs (1:500; Santa Cruz), rabbit anti-ATM (D2E2) (1:1000; Cell Signaling) or anti-pATM (S1981) (1:1000; Rockland). For pDNA-PKcs and DNA-PKcs, loading differences were visualized using rat anti-Lamin B (1:500; Santa Cruz); for pATM and ATM, α-tubulin (1:1000; Abcam Cambridge, GBR) served the same purpose. To visualize primary antibody binding, the membrane was probed with goat anti-rabbit conjugated with horseradish peroxidase (HRP) (1:15,000; Jackson Immunoresearch) or goat anti-mouse HRP (1:10,000; Abcam Cambridge, GBR). Bands were detected using a chemiluminescence detection kit (Amersham ECL Prime GE Healthcare Lifesciences Pittsburgh, PA, USA.

### DNA damage repair reporter system

Functional Non-homologous End Joining (NHEJ) or micro-homologous DNA damage repair were assayed using linearized pGL2-Luc vector (Promega Madison, Wisconsin, USA). The pGL2-Luc vector (100 ng) was linearized using either HindIII or EcoRI restriction enzymes (New England Biolabs Ipswich, Massachusetts, USA), and co-transfected with pRL-TK into U-118 MG cells using Lipofectamine 2000 (Invitrogen Carlsbad, California, USA). After 4 h, TTFields were applied for 24 h and cells were harvested and assayed for luciferase activity indicative of properly repaired and ligated plasmid reflecting DNA repair efficiency. The luciferase activity was measured using the Dual Luciferase Reporter Assay (Promega Madison, Wisconsin, USA) with the activity of the Renilla-Firefly luciferase serving as a control for transfection efficacy. The methods follow those reported by Wang et al. [18].

### Irradiation absorbance by TTFields ceramic transducer arrays – phantom models

To evaluate the absorption of the RT energy by TTFields transducer arrays – insulated ceramic arrays with hydrogel – identical to those in the Novo-TTF100A system used for the treatment of GBM patients – were placed on a solid-state phantom. The surface-to-source distance was 100 cm; field size was set to 10 × 10 cm. Dosimetry was assessed using a PTW Unidos Markus plane parallel ion chamber for high-energy electron measurements in solid-state phantoms (RPD Inc., Albertville, Minnesota, USA) connected to a PTW Unidos Webline (RPD inc, Albertville, Minnesota, USA). Doses of 2 Gy were applied using a 6 MV photon beam (Elekta Precise linear accelerator, Elekta Oncology Systems, Stockholm, Sweden) and dosimetry at the depths of 0, 20 and 60 mm below the arrays were measured. Absorption of the RT energy was tested perpendicular to the surface and other angles of attack were not evaluated.

### Skin effects of combined TTFields with RT

The animal studies were conducted at the Ben Gurion University of the Negev, Israel in compliance with “The Israel Animal Welfare Act” and following "The Israel Board for Animal Experiments" (approval no: IL-15-03-93). Sprague Dawley Rats were irradiated with 2 Gy, 5 times a week for 2 weeks using RadSource RS 2000 biological Research Irradiator (Suwanee, Georgia, USA) with a dose uniformity that exceeded 95%. Rats (*N* = 25) were divided into 5 groups (*n* = 5). In the control, Group 1 rats were irradiated without the arrays. In Groups 2-5, a pair of the arrays was placed 20 mm apart on the dorsal part of the abdomen. In order to test the effect of daily array replacement without irradiation on the skin, the arrays in Group 2 were replaced 5 times each week. In Group 3, the arrays were replaced twice a week similar to the clinical setting. In order to test the effect of irradiation through the arrays, rats in Group 4 were irradiated through the arrays 5 times a week and the arrays were replaced twice a week. In Group 5, the arrays were immediately placed right after RT and removed before the next RT cycle (Additional file 1: Table S1 shows the RT treatment and transducer replacement schedule). The rats were weighed throughout the study and average group body weights were calculated in grams. Skin conditions were visually evaluated at each replacement of the arrays according to the modified Draize scale (OECD 404: Acute Dermal Irritation/Corrosion http://www.oecd-ilibrary.org/environment/test-no-404-acute-dermal-irritation-corrosion_9789264070622-en).

At study termination, 12 days after treatment initiation, animals were weighed and then sacrificed by CO_2_ asphyxiation. Skin samples from the area under the arrays were marked, excised, fixed in 10% formalin, stained by H&E and evaluated. All slides were examined by a pathologist blinded to the treatment groups. Pictures were taken using a microscope (Olympus BX43) with 4×/10× objectives. Each skin sample was cut into 3-4 cross sections and evaluated using the following a semi-quantitative grading scale for inflammation, edema, hemorrhages and fibrosis as defined in Table 1.

**Table 1:** Grading scale for inflammation, edema, hemorrhages and fibrosis

Inflammation:	0 = No inflammatory infiltration.
	1 = Mild cellular infiltration with an increase of up to 10 cells per X10 HPF (high power field).
	2 = Moderate cellular infiltration with an increase of 10-20 cells per X10 HPF.
	3 = High cellular infiltration with an increase of 20-50 cells per X10 HPF.
	4 = Very high cellular infiltration with an increase of >50 cells per X10 HPF.
Necrosis:	0 = No necrosis.
	1 = Mild necrosis in the epidermis
	2 = Moderate necrosis in the epidermis
	3 = Severe necrosis in the epidermis
	4 = Severe necrosis in the dermis and in the epidermis
Edema:	0 = No edema.
	1 = Mild edema in the dermis
	2 = Moderate edema in the dermis
	3 = Severe edema in the dermis
	4 = Severe edema in the dermis and in the epidermis
Hemorrhages:	0 = No hemorrhages at all.
	1 = Mild hemorrhages in the dermis
	2 = Moderate hemorrhages in the dermis
	3 = Severe hemorrhages in the dermis
	4 = Severe hemorrhages in the dermis and in the epidermis
Fibrosis:	0 = Dermis shows no scar formation compared to normal skin.
	1 = Dermis shows very mild dermal fibrosis.
	2 = Dermis shows moderate dermal fibrosis.
	3 = Dermis shows high dermal fibrosis typical for scar formation.
	4 = Dermis shows high dermal fibrosis with typical scar tissue contraction.

### Statistical analysis

Unless stated otherwise, data are presented as mean ± SEM. Statistical significance of differences was assessed by 1-way ANOVA followed by Tukey’s range statistical test using GraphPad Prism 6 software (La Jolla, California, USA). Differences between all groups were compared, and were considered significant at values of 0.05 > *p > 0.01, **p < 0.01, and ***p < 0.001. All of the experiments were repeated at least three times.

## Results

### TTFields treatment enhances RT efficacy in glioma cells

**Fig. 1:**
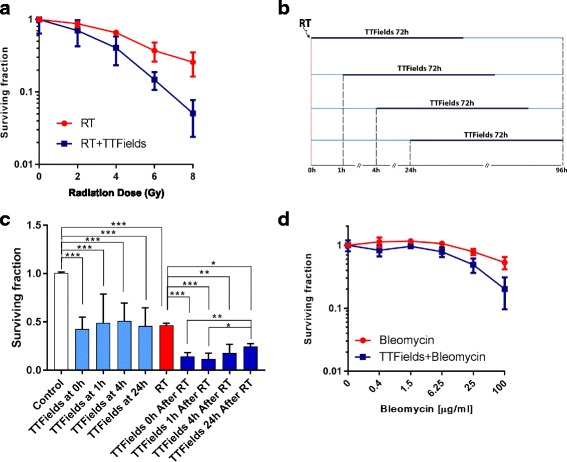
Surviving fraction of U-118 MG cells treated with 200 kHz TTFields (1.7 V/cm RMS) for 72 h (**a**). The efficacy of the combined treatment of TTFields and irradiation with 4 Gy was tested when 72 h TTFields treatment was applied immediately after RT or 1 h, 4 h, and 24 h after RT in U-118 MG cells (**b**). The efficacy of the combined treatment (**c**) of TTFields and irradiation with 4 Gy in U-118 MG cells RT (red column) and TTFields (pale blue column) treatments alone were compared with untreated cells (white column) – The combination treatment (dark blue column) was compared with RT alone (red column). Surviving fraction of U-118 MG cells treated with bleomycin alone or in combination with 200 kHz TTFields (1.7 V/cm RMS) for 72 h (**d**)

TTFields treatment alone (200 kHz, 1.7 V/cm RMS) for 72 h led to over 50% reduction in the surviving fraction of U-118 MG cells (Fig. 1c). The LN-18 cell line proved to be even more sensitive to TTFields as application of lower electric fields intensities (200 kHz, 1.0 V/cm RMS) led to 88 ± 6% reduction in the number of cells (Additional file 2: Figure S1A). In both cell lines, combining TTFields treatment and RT led to a further decrease in the surviving fraction at all radiation doses tested (Fig. 1a, Additional file 3: Figure S2A, B) in a synergistic manner (Table 2). In U-118 MG cells, delaying TTFields application for 24 h after RT (4Gy) (Fig. 1b), led to a reduction in treatment efficacy as compared to the effect of treatment initiated immediately and within 1 h after RT (Fig. 1c). The effect of TTFields on DNA damage-induced cytotoxicity was further studied in U-118 MG cells using bleomycin, a known inducer of single and double DNA strands breaks [19] (Fig. 1d). Similar to RT, TTFields potentiated bleomycin-induced cytotoxicity albeit with less magnitude (Table 2).

**Table 2:** Surviving fraction (SF) at tested radiation and bleomycin doses

Radiation _(Gy)_	SF _TTFields + RT_	SF_TTFields_ × SF_RT_	Radiosensitization effect
U-118 MG			
2	0.292	0.363	Synergism
4	0.169	0.273	Synergism
6	0.061	0.154	Synergism
8	0.021	0.107	Synergism
LN-18			
Radiation _(Gy)_	SF _TTFields + RT_	SF_TTFields_ × SF_RT_	Radiosensitization effect
2	0.025	0.054	Synergism
4	0.005	0.013	Synergism
6	0.001	0.004	Synergism
8	0.000	0.001	Synergism
U-118 MG			
Bleomycin _[μg/ml]_	SF _TTFields + Bleomycin_	SF_TTFields_ × SF_Bleomycin_	Chemosensitization effect
0.4	0.433	0.591	Synergism
1.5	0.500	0.606	Synergism
6.25	0.410	0.555	Synergism
25	0.255	0.411	Synergism
100	0.105	0.277	Synergism

### TTFields delays repair of RT-induced DNA damage

**Fig. 2:**
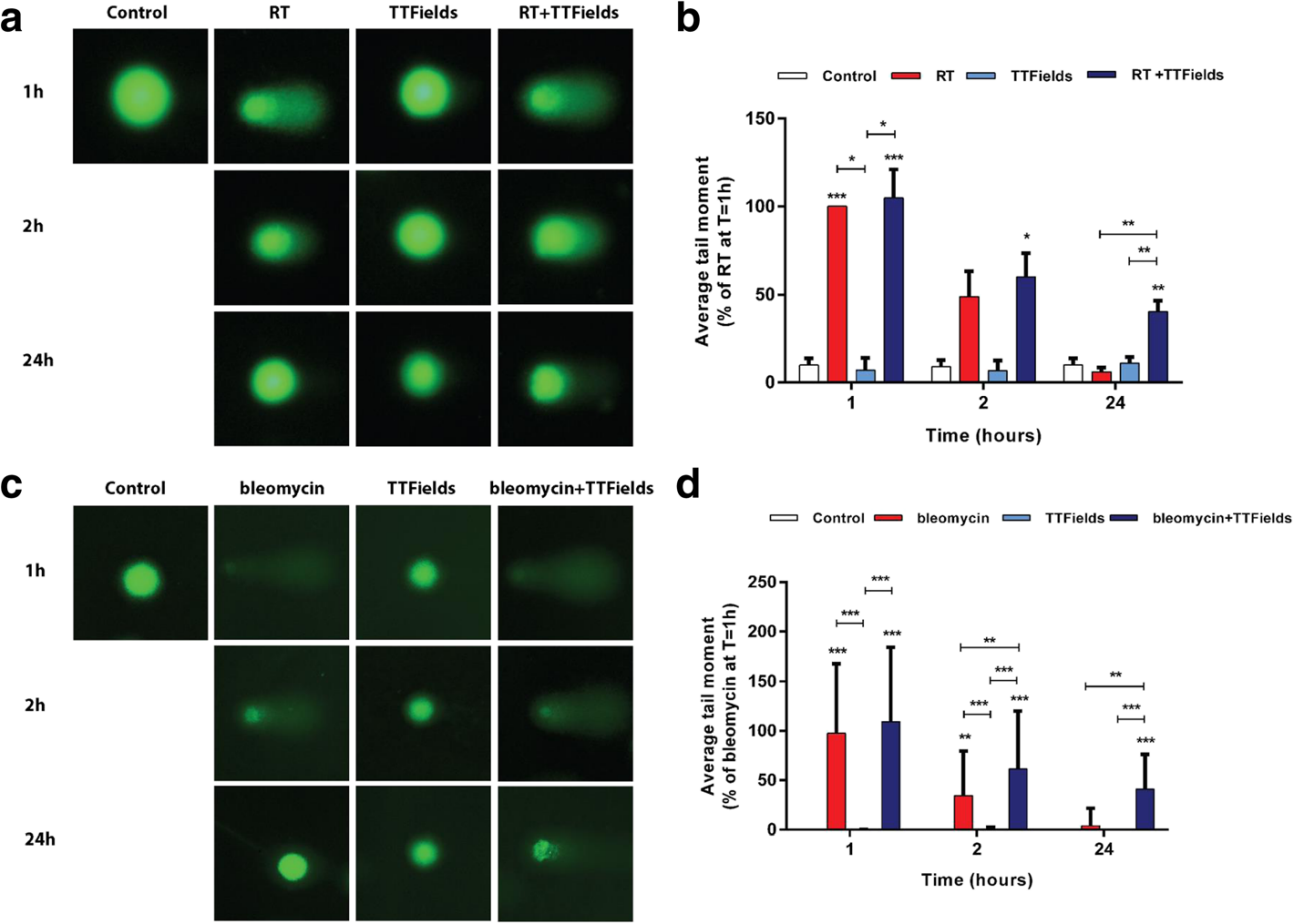
TTFields Delay Irradiation-Induced DNA Damage Repair in glioma cells. U-118 MG cells were irradiated with 4 Gy and immediately treated with TTFields applied for 1 h, 2 h or 24 h (**a**-**b**) or treated with bleomycin followed by TTFields application (**c**-**d**). Effect on DNA repair was measured as tail moment in the comet assay

RT-induced cytotoxicity is dependent on cellular DNA double strand break repair. We therefore analyzed if TTFields influenced DNA repair capacity of RT-inflicted DNA damage by assessing unrepaired DNA using the alkaline comet assay (Fig. 2, Additional file 3: Figure S2). In U-118 MG cells treated with TTFields within 1-2 h after RT, the average tail moment was similar to that observed following RT alone (Fig. 2a, b). While the majority of the initial DNA damage was repaired within 24 h after RT alone, more than 40% of the initial DNA damage remained unrepaired when TTFields were subsequently applied demonstrating that TTFields impaired cellular DNA repair capacity. Similar results were obtained with LN-18 cells, though the inhibition of the repair capacity was already detected after 2 h of TTFields application (Additional file 3: Figure S2A, B). Comet assays performed on bleomycin-treated U-118 MG cells also revealed that TTFields significantly halted DNA repair, further illustrating an effect on DNA repair by TTFields (Fig. 2c-d).

**Fig. 3:**
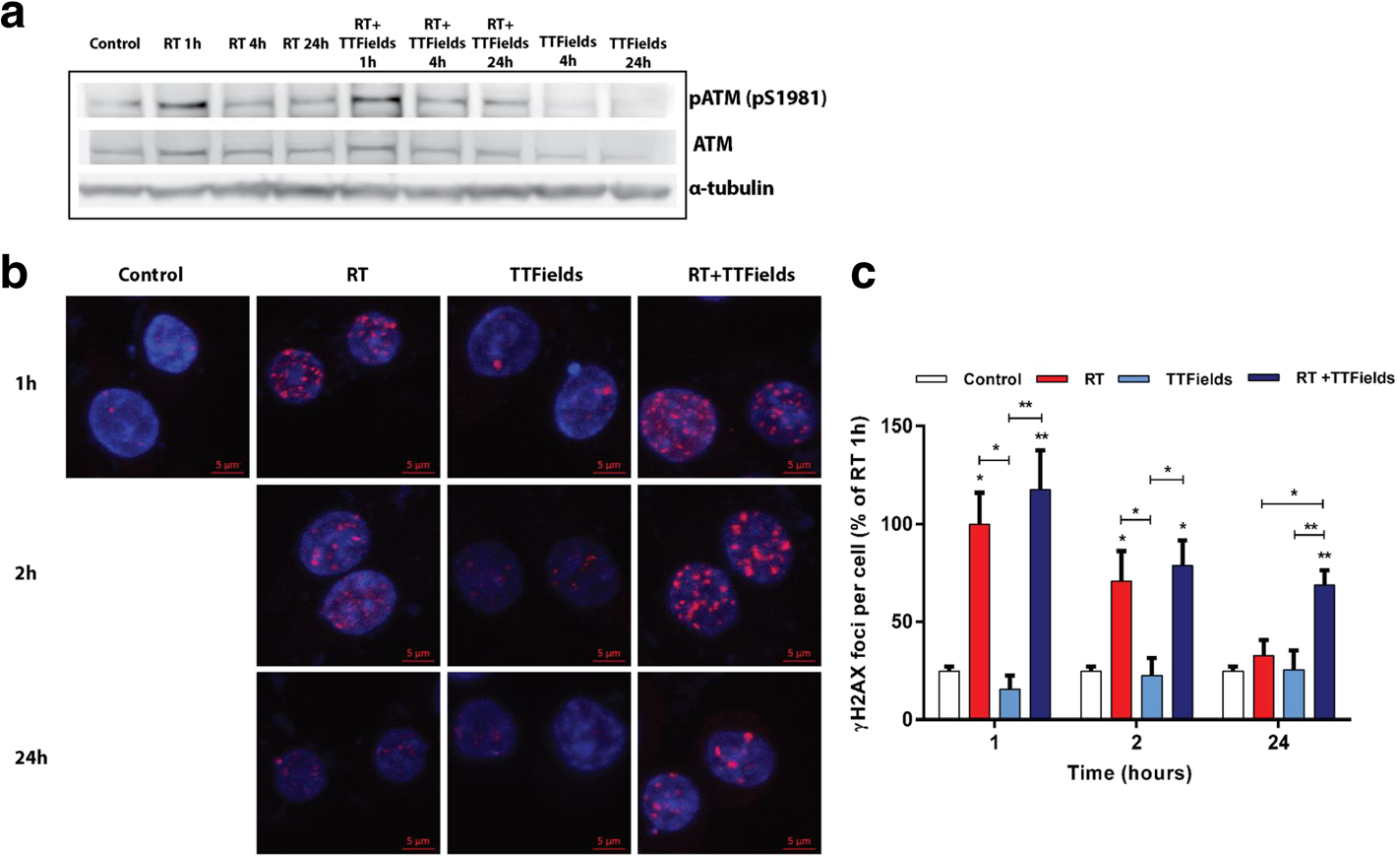
TTFields Treatment after RT causes the Retention of γH2AX Foci formation. **a**. U-118 MG cells were irradiated with 4 Gy RT and immediately treated with TTFields for 1 h, 2 h, or 24 h. **a** pATM (pS1981) or total ATM expression was examined by immunoblotting with α-tubulin used to confirm equal loading. **b**-**c** Effect of RT (4 Gy), TTFields or their combination on formation and resolution of γH2AX foci was analyzed by immunofluorescence with DAPI used for counterstaining of cell nuclei. Scale bar - 5 μm. In (**c**) the average γH2AX foci in cells with more than 5 foci were quantified

To test if the observed delayed DNA repair was the result of a failure in the early steps of the repair machinery, we examined if TTFields influenced the phosphorylation status of ATM, one of the earliest activators triggered in response to DNA DSBs. However, a similar expression and activation pattern of ATM was found in irradiated U-118 MG cells with or without TTFields (Fig. 3a). Noteworthy, TTFields alone led to a decrease in total ATM expression level and S1981 phosphorylation pattern. Phosphorylation of histone variant H2AX is also a marker of DNA DSBs and is one of the early steps required for the assembly of DNA repair proteins as well as for activation of checkpoints proteins [20]. As in the case of pATM, there were no major differences in the total amount of the γH2AX foci per cell at 1 h or 2 h after the combined treatment compared with RT alone (Fig. 3b and c). In contrast, at 24 h the amount of residual foci was higher in RT + TTFields treated cells as compared to either RT or TTFields treatment alone (Fig. 3c). Thus, these results support the blocking effect of TTFields on delayed cellular DNA repair of RT-induced DNA damage.

**Fig. 4:**
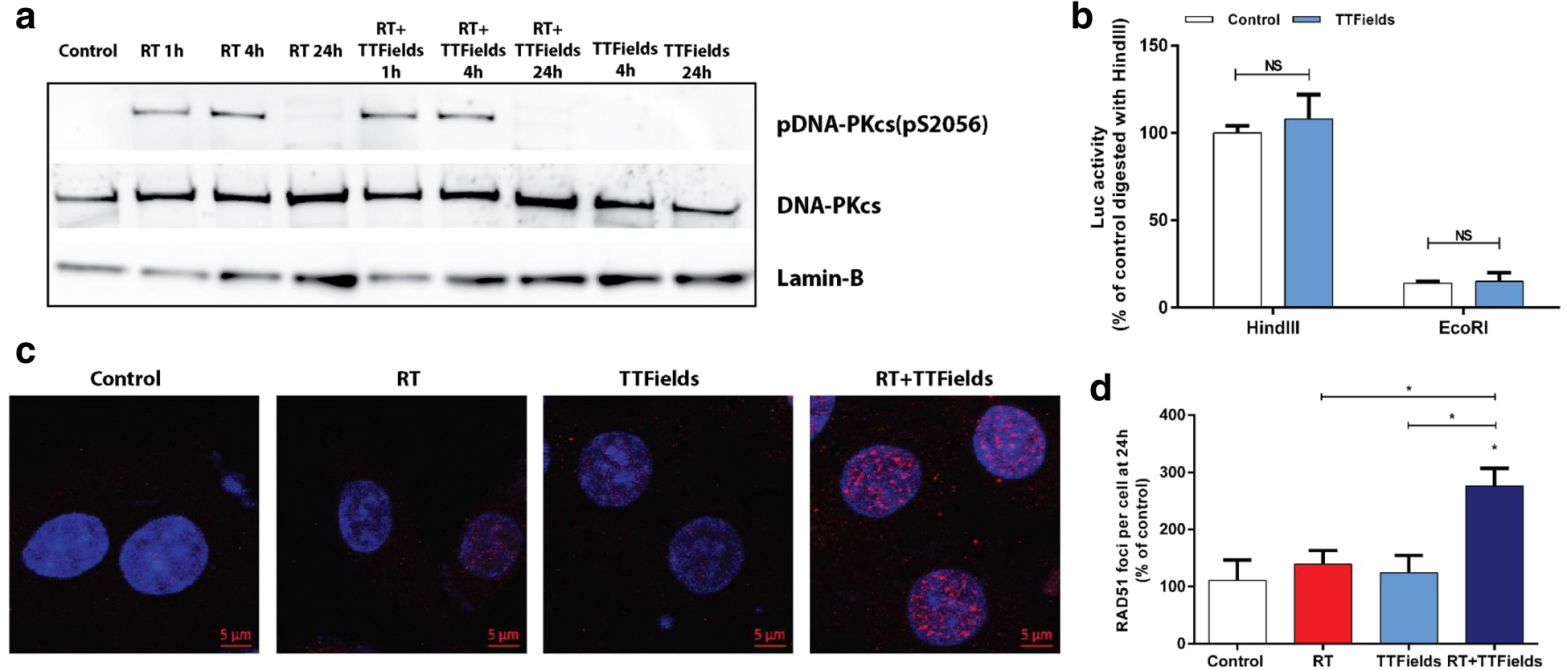
TTFields Influence DNA Damage Repair by Homologous Recombination in Glioma Cells. **a** pDNA-PKcs (pS2056) and total DNA-PK were compared between U-118 MG cells either untreated or treated with RT or TTFields alone or their combination at indicated time points post RT (4 Gy). Lamin B was used as loading control. **b** U-118 MG cells were transfected with an intact pGL2-Luc vector or vector that was linearized with either HindIII or EcoRI. Luc activity was measured in cells prior and post 24 h TTFields treatment. **c**-**d** U-118 MG cells were irradiated with 4 Gy and immediately treated with TTFields for 1 h, 2 h, or 24 h. **c** Rad 51 foci formation was analyzed by immunofluorescence at 24 h post treatment. Rad 51 foci (Red) and DAPI (blue) stained nuclei are shown. Scale bar - 5 μm. **d** The average Rad51 foci in cells with more than 5 foci are shown

Autophosphorylation of the DNA-PK catalytic subunit, DNA-PKcs, is an indirect measurement for DNA-PKcs activity, a central component in non-homologous end joining (NHEJ). Indeed, a clear increase in phosphorylation of DNA-PK (pS2056) at 1 h and 4 h post RT was observed after RT with similar increase observed when TTFields were applied after RT (Fig. 4a). To further understand if NHEJ was involved in the effect of TTFields on cellular DNA repair, a plasmid-end joining assay with linearized pGL2-Luc vectors was used for U-118 MG cells treated with TTFields for 24 h (Fig. 4b). In U-118 MG cells transfected with pGL2-Luc vectors linearized with either HindIII or EcoRI, no difference in ligation of the vectors was evident between untreated cells or cells exposed to TTFields for 24 h (Fig. 4b). Next, we tested whether the homologous recombination (HR) pathway was affected by TTFields by analyzing Rad51 foci formation in single and combined treated cells. As seen in Fig. 4c and d, when TTFields were applied for 24 h after RT, Rad51 foci formation was increased as compared to RT alone. The results support that TTFields influence cellular DNA repair capacity by altering the HR repair pathway.

### Irradiation absorbance by TTFields ceramic transducer arrays – phantom models

**Fig. 5:**
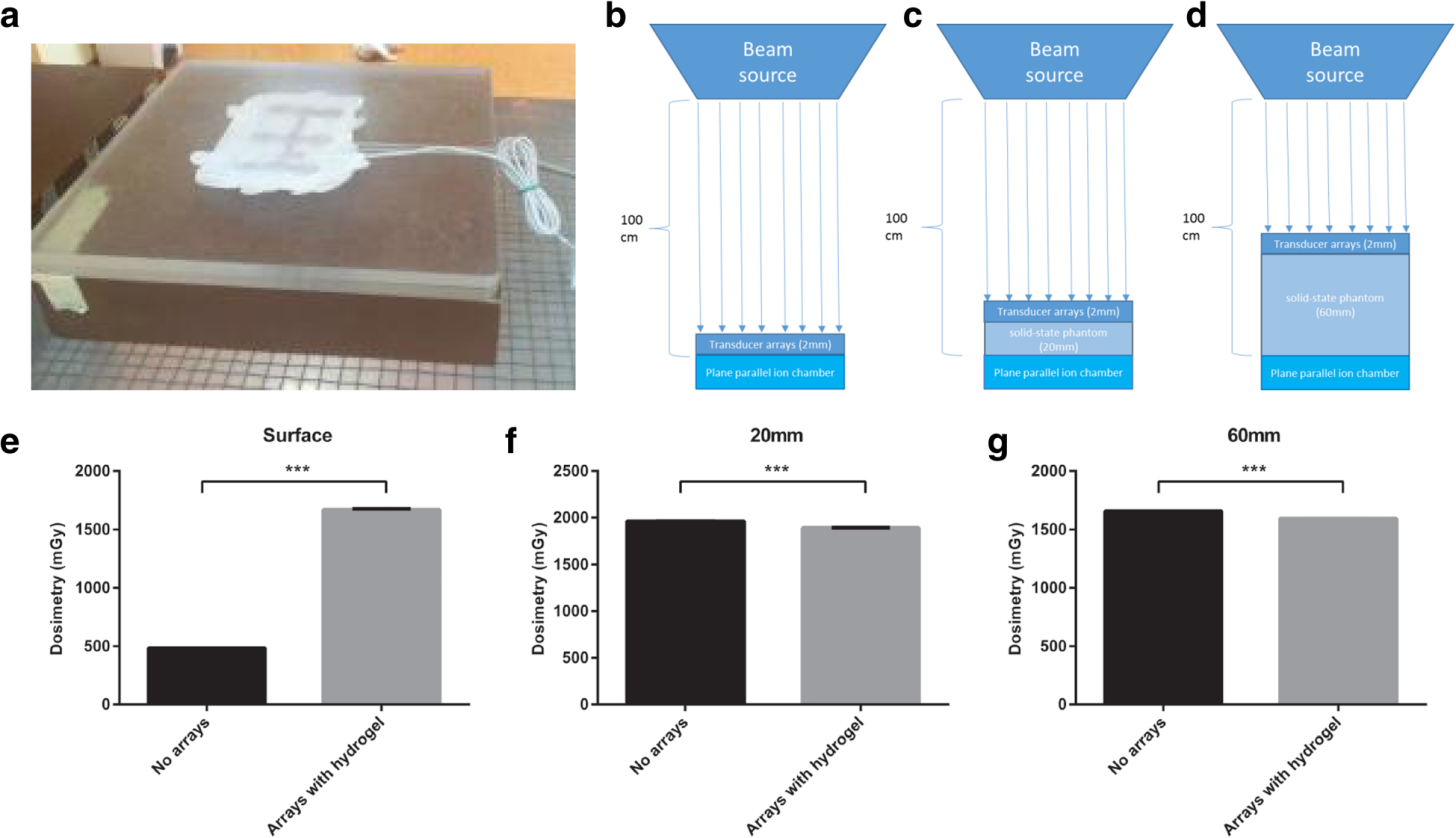
Irradiation Absorbance by TTFields Ceramic Transducer Arrays. Insulated ceramic arrays with hydrogel were placed on a solid-state phantom (**a**). Dosimetry was measured at the depths of 0 mm (**b**), 20 mm (**c**), and 60 mm (**d**) – dimensions in the figure are not to scale. Dosimetry of RT in phantom with or without ceramic TTFields arrays at the phantom surface (**e**), and at 20 mm (**f**) and 60 mm (**g**) are given

Results from the cell culture experiments suggest that concurrent treatment with RT and TTFields may have clinical utility. One option for combining TTFields with RT would be to irradiate through the TTFields transducers in order to minimize the need to remove and replace arrays. Therefore there is a need to understand RT dose distribution in such an approach. Dosimetric measurements preformed in phantom models (Fig. 5a-d), with or without the transducers, demonstrated a dramatic increase in the doses received just below the arrays as compared to the control (Fig. 5e; 1670 ± 4 vs. 486 ± 1 mGy, respectively; ***P < .001, Student t-test). This reflected an increase of 344% in the RT dosage at the phantom surface. The presence of the ceramic arrays led to a significant yet minor reduction (<4%) in the RT intensities received at the depth of 20 mm and 60 mm below the arrays as compared to the control (Fig. 5f-g; ***P < .001, Student t-test). These results suggest that while RT dose applied to the tumor may be minimally affected by the presence of the arrays, the skin below the arrays is expected to receive much higher RT dosages and thus may be subject to increased risk.

### Effects of combined TTFields with RT on the skin

**Fig. 6:**
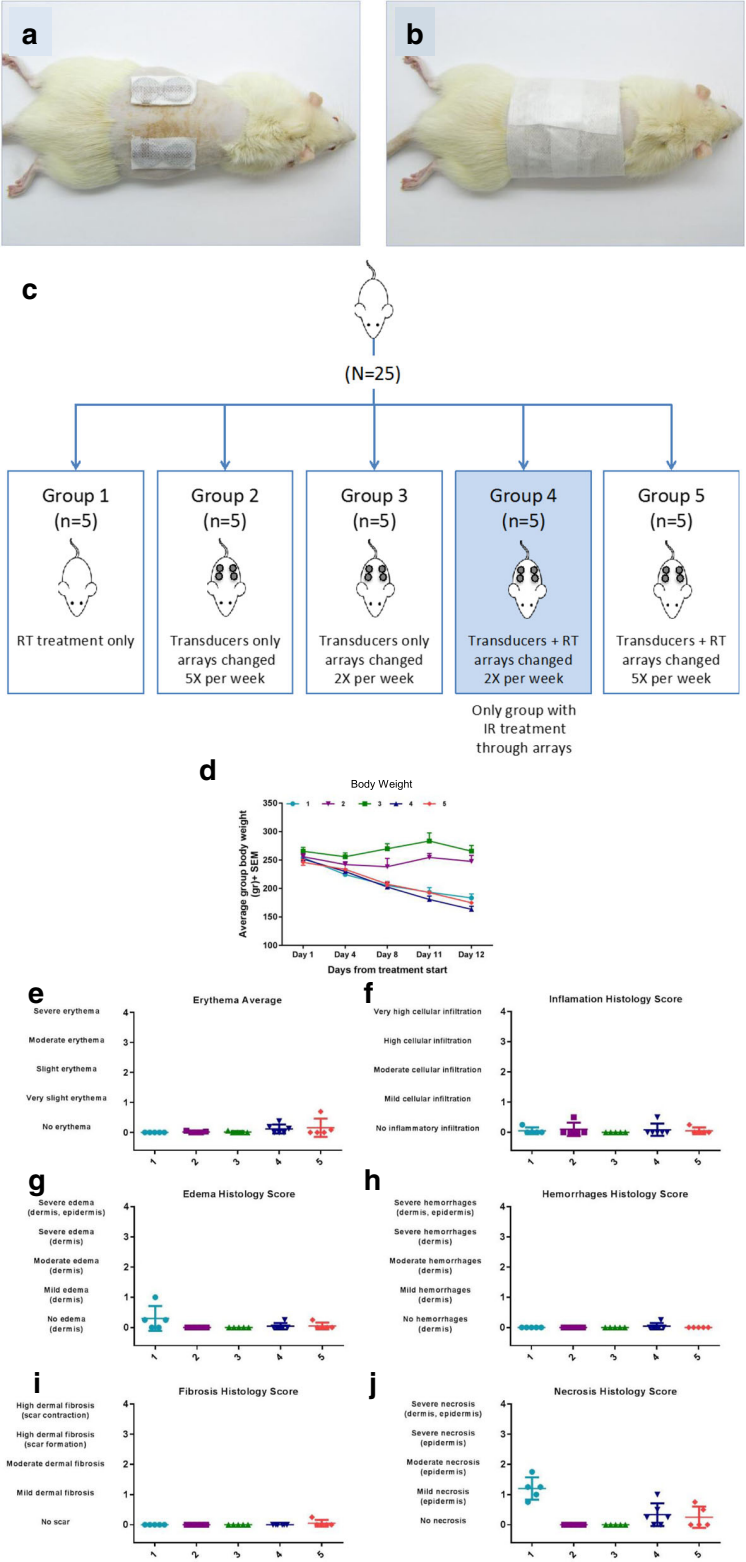
Effect of low dose RT through TTFields ceramic transducer arrays on the rat skin. Ceramic transducer arrays placed on the rat’s dorsal skin (6**a**-**b**). The five treatment groups are shown in 6**c**, Groups 4 and 5 received RT. Effect of TTFields and RT on the weight of non-tumor bearing rats (**d**). The group average body weight (g) over 2 weeks are shown. Figures **e** through **j** show the gross histological assessment of erythema (**e**) and microscopic histology assessment of inflammation (**f**), edema (**g**), hemorrhages (**h**) and fibrosis (**i**). Mild (Score 1) to moderate (Score 2) necrosis was observed in the epidermis of all RT groups (**j**). For scoring criteria please see Table 1

To determine if the ceramic array devices of TTFields influenced RT tissue toxicity, rats were irradiated with or without transducer arrays placed on the skin (Fig. 6a-c). The body weight for the irradiated groups was significantly different compared to the non-irradiated groups starting on Day 8 (Fig. 6d). There was no significant body weight loss associated with the presence of the arrays among irradiated animals.

Visual inspection of the skin revealed very slight erythema in rats with arrays being irradiated (Groups 4 and 5) (Fig. 6e). No visual effect was observed on skin edema (Results not shown). Histology analysis revealed no effect on: inflammation (Fig. 6f), edema (Fig. 6g), hemorrhages (Fig. 6h) and fibrosis (Fig. 6i). Mild (Score 1) to moderate (Score 2) necrosis was observed in the epidermis of all RT groups (Fig. 6j). The rats that were irradiated without arrays (Group 1) demonstrated the highest increase in dermal necrosis compared to all other groups.

## Discussion

Glioblastoma is the predominating malignant brain tumor in adults and despite aggressive therapy with combined radio-and chemo-therapy treatment (e.g. fractionated irradiation at a dose of 2 Gy with concomitant daily TMZ chemotherapy) [5, 6], GBM patients rarely respond to treatment and have poor long-term survival. One possibility for enhancing the RT effect is by inhibition or delay of DNA repair following RT, thereby promoting cell death. The effectiveness of such a strategy was recently demonstrated by combining pharmacological inhibitors of DDR pathways (i.e. cell-cycle checkpoints and the DNA damage repair) with standard RT [21, 22]. This is of particular relevance for the treatment of malignant glioma tumors that are highly resistant to therapies that inflict DNA damage [23, 24].

TTFields are known to disrupt normal mitosis through the depolymerization of microtubules and interruption of the spindle structure leading to mitotic catastrophe and the formation of non-viable daughter cells [12–15]. Here, we report that besides their anti-mitotic properties, TTFields applied after RT may serve as a RT potentiating strategy for glioma. We show a synergistic effects for RT and TTFields when applied to U-118 MG and LN-18 glioma cells. Moreover, we show that application of TTFields inhibited the repair of RT- or chemically-induced DNA damage possibly by blocking homologous recombination repair.

Our data also demonstrate that TTFields treatment without RT results in more than 50% and 88% reduction of surviving fraction in U-118 MG (MGMT methylated) and LN-18 cells (MGMT un-methylated) respectively, providing further evidence of TTFields’ efficacy against glioma cells regardless of the MGMT methylation status. ‘Classic’ radiosensitizers (e.g. misonidazole, bromodeoxyuridine) usually do not show inherent cytotoxic activity. However, similar to TTFields, commonly used radiosensitizers (e.g. taxanes, cisplatin, 5-fluorouracil [5-FU]) possess an inherent ability to inflict cell death [1].

Recently it was reported that TTFields application before RT led to decreased glioma cell survival as a result of increased mitotic catastrophe and blockade of DDR [17]. Of note, our results are in line with these reports despite the difference in the order of TTFields and RT application between the two studies, as both studies demonstrate synergism between TTFields and RT. The similarity between the results obtained when TTFields were applied prior to or after RT suggests a common mechanism of action underlying TTFields’ effect on DDR. There are several possible mechanisms that may account for these results.

One such mechanism is the inhibition of one or more DNA repair pathways by TTFields, which become more prominent with longer exposure to TTFields. When TTFields were applied after RT, the early steps of the DNA damage recognition and repair process were unaffected as evident from our results from γH2AX foci counts and ATM phosphorylation, while application of TTFields prior to RT led to increased damage within an hour from irradiation [17]. Similarly, TTFields did not affect the rapid, fast occurring NHEJ response, as evident from the lack of effect on RT-induced DNA-PKcs phosphorylation pattern and also from results of the plasmid rejoining assay. However, we observed a significant increase in the RAD51 foci number in cells treated with TTFields for 24 h after RT, suggesting a blockade in homologous recombination repair pathway, which could in part be responsible for the observed increase in DNA damage and reduced cell survival after combined treatment.

Ionizing radiation causes arrest of mammalian cells in the G1 and G2 phases, thereby decreasing the probability of cells to be affected by anti-mitotic modalities such as TTFields [25–27]. However, our data suggest synergistic efficacy for RT and TTFields treatment, implying that the benefits of this combination overcome such possible drawbacks. Future studies are required to pinpoint the components and processes that are affected.

In the EF-14 phase 3 clinical trial, TTFields applied during the course of TMZ maintenance, demonstrated a significant increase in progression-free survival and overall survival as compared to patients treated with TMZ alone [7]. TMZ, is an alkylating agent leading to the formation of O6-methylguanine, which results in the generation of DNA single- and DSBs [28]. The results demonstrated herein and by Kim et al. [17], offer a new perspective on the EF-14 trial positive outcomes based on the synergism between TTFields and DNA damaging modalities such as TMZ.

The synergistic effect of TTFields and RT observed when the electric fields were applied prior to or after irradiation, suggests GBM patients may benefit from the concomitant application of TTFields with daily-fractionated irradiation. From a practical point of view, this would mean either daily removal of the TTFields transducers prior to radiotherapy or irradiating through the arrays. In order to test the feasibility of the latter option, we measured the amount of RT dose absorbed by the arrays placed over a phantom model. Our results show that while the RT doses in deep tissue are minimally affected by the presence of the arrays (<4% reduction in RT intensity at 20 and 60 mm below the arrays), the dosimetry measurements indicate that the energy buildup starts just below the arrays, which may jeopardize the “skin sparing” effect. Since both approaches, either irradiating through the transducer arrays or daily removal of the arrays, could increase the risk of skin toxicity, we tested for dermatological adverse effects in a rat model. Skin screening revealed that RT administered through the ceramic transducer arrays did not lead to adverse skin reactions such as edema, inflammation, hemorrhages or fibrosis. As expected, dermal necrosis was increased in all irradiated rats. Nevertheless, it is also important to regard the potential of late-onset effects of exposure to RT. Although no marked effects were detectable after irradiating through the transducer arrays, there is still a remaining possibility for long-term effects. Clinical studies are therefore under development to explore the effect of external beam RT when applied through the transducer arrays in conjunction with active TTFields on human skin.

The approach of combining RT and TTFields could potentially benefit tumor types other than GBM. Moreover, studies examining the combination of TTFields with chemotherapies that inflict secondary DNA DSBs (e.g. anthracyclines, platinum compounds) will be of high interest [29, 30]. As TTFields are locally applied to the tumor region, these combination treatments could assist in focusing the systemic effect of such cytotoxic agents to mimic the local tumor control of RT. This approach could be advantageous for cases in which RT cannot be applied due to risk of local tissue toxicity.

## Conclusions

Taken together, our results provide a strong preclinical rationale for starting TTFields application before and immediately after RT to improve efficacy outcomes for GBM patients. The results of this preclinical study indicate that this could be accomplished by leaving the transducer arrays attached to the patients’ skin during radiation therapy. Accordingly, it would be of interest to evaluate the impact of direct irradiation through the TTFields ceramic transducer arrays in a clinical trial setting.

## Additional files

Additional file 1: Table S1. RT treatment schedule and transducer replacement schedule. Rats received RT on days 0-4 and 7-11 (indicated by V). Groups 4 and 5 had arrays placed on their dorsal surface (arrays where replaced days indicated by V). Rats where euthanized on day 12. (DOCX 96 kb)

Additional file 2: Figure S1. TTFields effect on LN-18 glioma cells. Surviving fractions of LN-18 cells treated with TTFields (200 kHz, 1.0 V/cm) for 72 h either alone or immediately after irradiation with 4 Gy (A). Surviving fraction of LN-18 cells treated with RT alone or with RT at various doses followed by 200 kHz TTFields (1.0 V/cm RMS) for 72 h. Results of the combined treatments were normalized to the effect of TTFields alone (B). (PPTX 2783 kb)

Additional file 3: Figure S2. TTFields Delay Irradiation-Induced DNA Damage Repair in glioma cells. LN-18 cells were irradiated with 4 Gy RT and immediately treated with TTFields applied for 1 h, 2 h or 24 h (A-B). Effect on DNA repair was measured as tail moment in the comet assay. (PPTX 1940 kb)

## Abbreviations

ATCC: American type culture collection
ATM: Ataxia Telangiectasia Mutated
DAPI: 4′,6-diamidino-2-phenylindole
DSBs: Double strand breaks
GBM: Glioblastoma multiforme
Gy: Gray unit
HR: Homologous recombination
PKcs: protein kinase, catalytic subunit
PMN-PT: Lead Magnesium Niobate–lead Titanate
RIPA: Radioimmunoprecipitation assay buffer
RMS: Root-mean-square
RT: Radiation treatment
TBST: Tris-buffered saline (TBS) and Polysorbate 20
TMZ: Temozolomide
TTFields: Tumor treating fields
