# Supplementary material for: Tumor treating fields (TTFields) delay DNA damage repair following radiation treatment of glioma cells
Source: Radiat Oncol. 2017 Dec 29;12:206. doi: 10.1186/s13014-017-0941-6 (PMC5747183; doi:10.1186/s13014-017-0941-6)
Supplement: Supplementary file 1 — RT treatment schedule and transducer replacement schedule. Rats received RT on days 0-4 and 7-11 (indicated by V). Groups 4 and 5 had arrays placed on their dorsal surface (arrays where replaced days indicated by V). Rats where euthanized on day 12. (DOCX 96 kb) [file 13014_2017_941_MOESM1_ESM.docx]

**Supplemental Table 1.** RT treatment schedule and transducer replacement schedule. Rats received RT on days 0-4 and 7-11 (indicated by V). Groups 4 and 5 had arrays placed on their dorsal surface (arrays where replaced days indicated by V). Rats where euthanized on day 12.

**
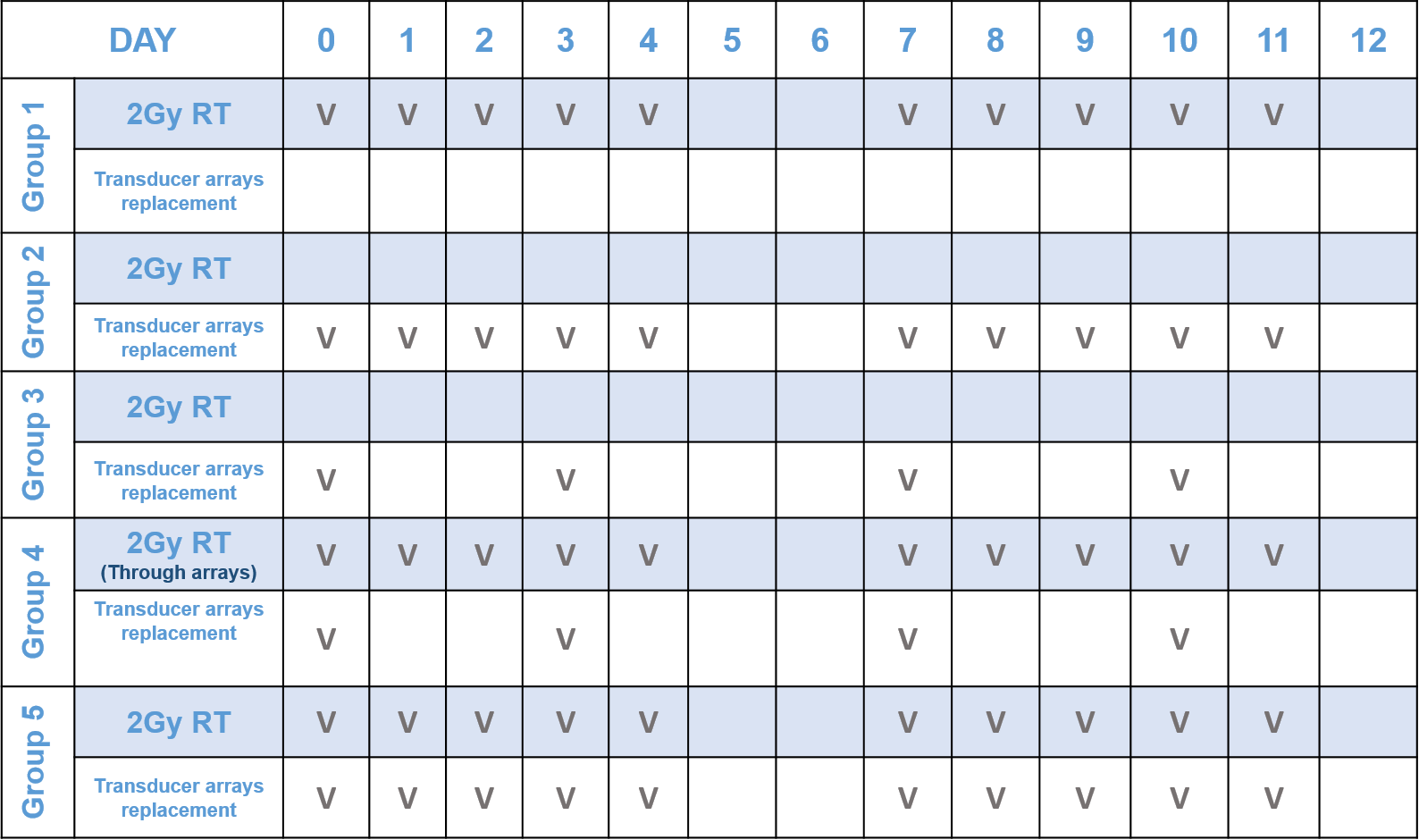
**
