# Supplementary figures and images for: Tumor treating fields (TTFields) delay DNA damage repair following radiation treatment of glioma cells
Source: Radiat Oncol. 2017 Dec 29;12:206. doi: 10.1186/s13014-017-0941-6 (PMC5747183; doi:10.1186/s13014-017-0941-6)

## Slide 1
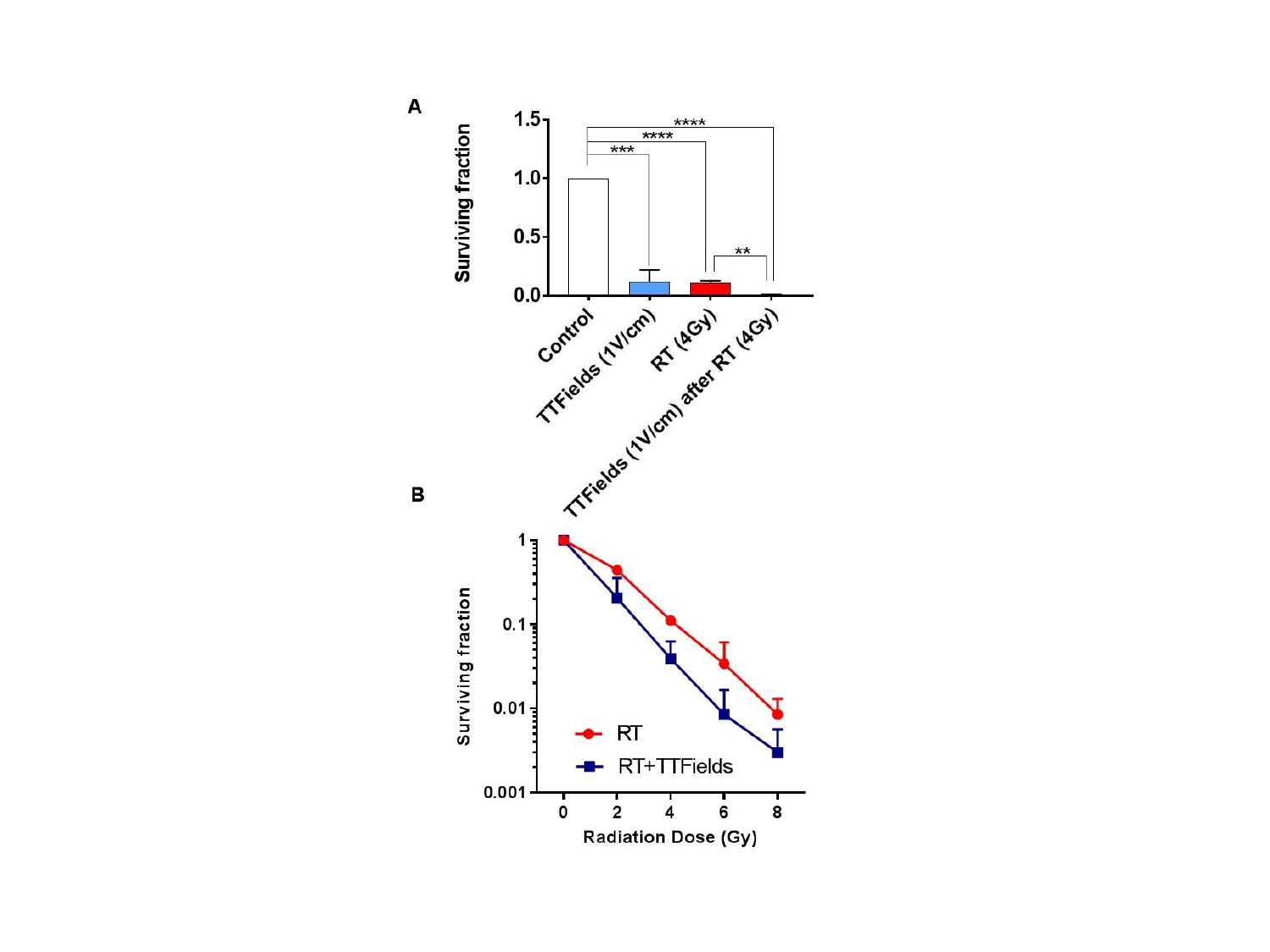

Supplement: Supplementary file 2 — TTFields effect on LN-18 glioma cells. Surviving fractions of LN-18 cells treated with TTFields (200 kHz, 1.0 V/cm) for 72 h either alone or immediately after irradiation with 4 Gy (A). Surviving fraction of LN-18 cells treated with RT alone or with RT at various doses followed by 200 kHz TTFields (1.0 V/cm RMS) for 72 h. Results of the combined treatments were normalized to the effect of TTFields alone (B). (PPTX 2783 kb) [file 13014_2017_941_MOESM2_ESM.pptx]

## Slide 1
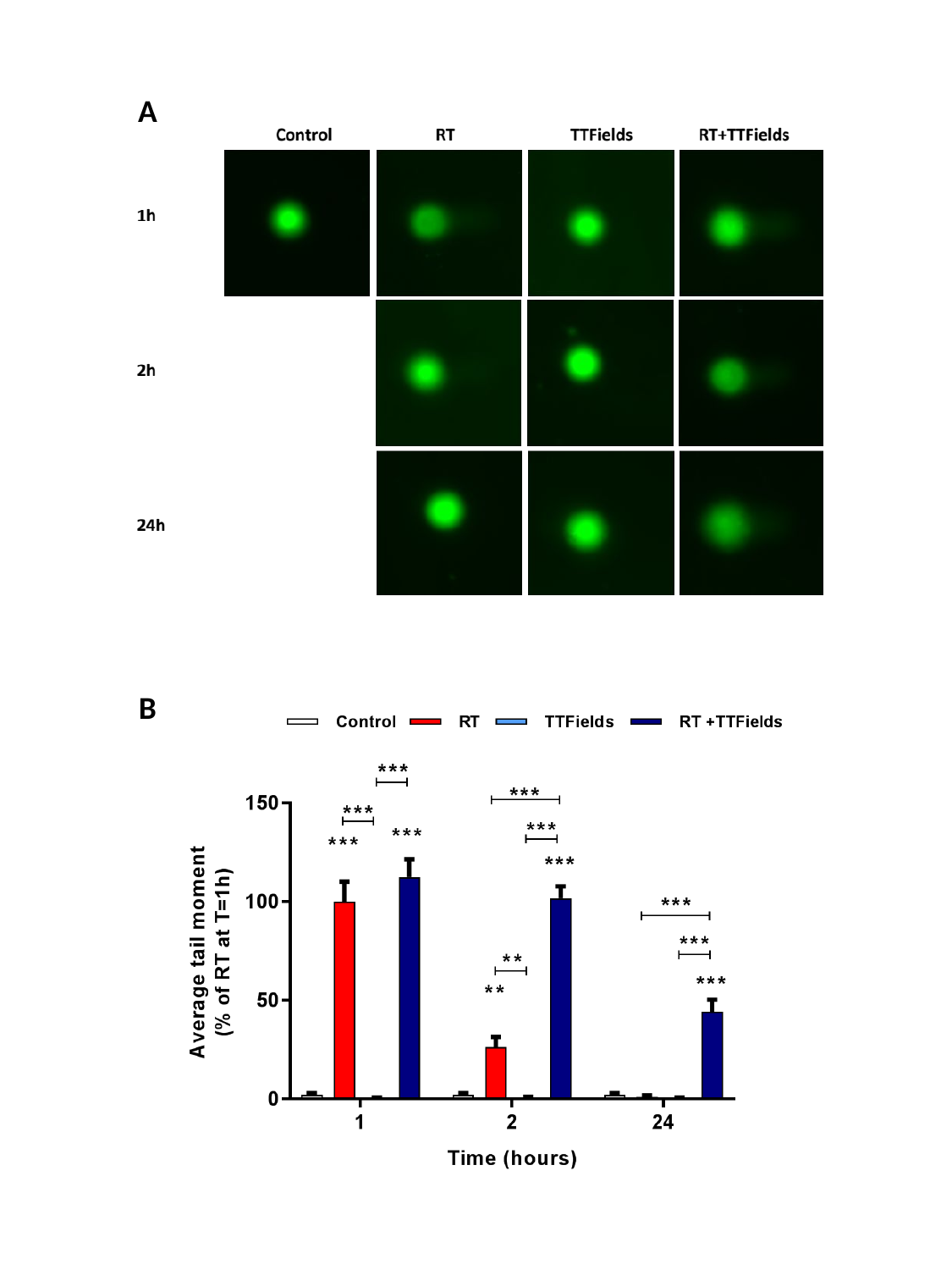

A
B

Supplement: Supplementary file 3 — TTFields Delay Irradiation-Induced DNA Damage Repair in glioma cells. LN-18 cells were irradiated with 4 Gy RT and immediately treated with TTFields applied for 1 h, 2 h or 24 h (A-B). Effect on DNA repair was measured as tail moment in the comet assay. (PPTX 1940 kb) [file 13014_2017_941_MOESM3_ESM.pptx]
